# Endoscopic endonasal resection of the cavernous sinus medial wall for functioning pituitary adenomas: A propensity score-matched retrospective cohort study

**DOI:** 10.1097/MD.0000000000049633

**Published:** 2026-07-03

**Authors:** Mengyang Xing, Jing Wang, Meng Li, Jinxia Liu, Yingjiang Xu, Qishuai Liu, Donghai Wang, Pengfei Liu

**Affiliations:** a Department of Neurosurgery, Shandong Medical and Pharmaceutical University Hospital, Binzhou, Shandong, China; b Department of Neurosurgery, Qilu Hospital, Shandong University, Jinan, Shandong, China; c Department of Ultrasonic, Shandong Medical and Pharmaceutical University Hospital, Binzhou, Shandong, China; d Department of Pain Treatment, Shandong Medical and Pharmaceutical University Hospital, Binzhou, Shandong, China; e Department of Interventional Vascular Surgery, Shandong Medical and Pharmaceutical University Hospital, Binzhou, Shandong, China.

**Keywords:** biochemical cure, cavernous sinus medial wall, functioning pituitary adenomas, propensity score matching, tumor recurrence

## Abstract

Functioning pituitary adenomas (FPAs) account for 63% to 85% of pituitary adenomas. Although endoscopic endonasal surgery targets biochemical remission and reduced recurrence, cavernous sinus medial wall (CSMW) invasion often leads to incomplete resection and persistent disease. This retrospective study evaluated the clinical value of combining tumor excision with CSMW resection in patients with FPAs. We compared 60 patients who underwent tumor excision plus CSMW resection with contemporaneous controls who underwent tumor excision alone. Propensity scores based on age, sex, body mass index, Knosp grade, hormone subtype, and tumor size/volume were used for 1:1 nearest-neighbor matching, yielding 60 pairs. In the matched cohort, the CSMW resection group achieved higher sustained biochemical cure (≥6 months) (85.0% vs 61.7%) and clinical symptom relief (83.3% vs 56.7%), and a higher magnetic resonance imaging-defined gross total resection rate (95.0% vs 81.7%), with a lower recurrence rate (3.3% vs 15.0%) (all *P* < .05). After multivariable adjustment (age, sex, body mass index, and Knosp grade), CSMW resection remained independently associated with sustained biochemical cure (odds ratio [OR] = 4.77, 95% confidence interval [CI] 1.83–12.43), symptom relief (OR = 4.68, 95% CI 1.79–12.21), and gross total resection (OR = 4.55, 95% CI 1.04–19.95), and with reduced recurrence (OR = 0.16, 95% CI 0.03–0.78). Postoperative cerebrospinal fluid leakage and intracranial infection were less frequent with CSMW resection (both 3.3% vs 18.3%; OR = 0.13, 95% CI 0.03–0.54), whereas length of stay and other sinonasal complications did not differ materially. Tumor excision combined with CSMW resection may improve key clinical outcomes in FPAs and warrants consideration in appropriately selected patients.

## 1. Introduction

Pituitary adenomas (PAs) are common neuroendocrine tumors arising from cells of the adenohypophysis.^[1–3]^ They account for approximately 10% of all intracranial neoplasms and are detected in 8.2% to 14.7% of the general population, whereas autopsy series report prevalences as high as 30%.^[4]^ PAs can cause substantial morbidity through excessive hormone secretion, compression of the optic apparatus, cranial nerves and adjacent structures, disruption of hypothalamic–pituitary endocrine function, and acute complications such as pituitary apoplexy and irreversible visual loss.^[5,6]^ Clinically, PAs are broadly classified as functional pituitary adenomas (FPAs) and non-FPAs, according to whether they are associated with clinically evident hormone hypersecretion.^[6,7]^

For FPAs, the primary therapeutic goals are postoperative biochemical cure, symptom relief, and prevention of tumor recurrence. However, traditional management with simple tumor excision alone often fails to fully achieve these goals because many FPAs invade the parasellar dura. Anatomically, the cavernous sinus medial wall (CSMW) is a thin dural interface between the pituitary gland and the cavernous sinus that forms a key boundary for parasellar extension. Increasing evidence suggests that tumor invasion of the CSMW can be occult under endoscopic visualization and and may contribute to microscopic residual disease despite apparent gross total resection. Accordingly, the CSMW represents one of the most vulnerable portions of the parasellar region.^[8,9]^

Therefore, achieving durable biochemical remission in FPAs may depend on tumor excision combined with resection of the cavernous sinus medial wall. This propensity score-matched retrospective cohort study aimed to evaluate the clinical efficacy and safety of this combined approach in patients with FPAs.

## 2. Methods and materials

### 2.1. Trial design

We retrospectively reviewed patients with FPAs treated between October 1, 2021 and December 1, 2023. The CSMW resection group consisted of 60 consecutive patients who underwent tumor excision combined with resection of the CSMW. The control pool comprised 200 patients who underwent tumor excision alone and served as candidates for the non-CSMW resection group. To reduce baseline confounding, propensity scores for receiving CSMW resection were estimated using a logistic regression model based on age, sex, Knosp grade, hormone subtype, body mass index (BMI), and tumor size/volume category. Patients were then matched 1:1 using nearest-neighbor propensity score matching to select 60 patients from the control pool, yielding 60 matched pairs for outcome comparison. Baseline characteristics were evaluated before and after matching to assess covariate balance.

Inclusion criteria included: primary surgery for a pituitary adenoma during the study period; functioning pituitary adenoma confirmed by biochemical evidence of hormone hypersecretion and postoperative histopathology; and endoscopic endonasal transsphenoidal approach performed by the same surgical team. Evidence of CSMW involvement on preoperative magnetic resonance imaging (MRI) and/or intraoperative findings documented before any CSMW manipulation. Exclusion criteria were: previous pituitary surgery or radiotherapy; nonfunctioning pituitary adenoma; other sellar/parasellar lesions; and non-endoscopic endonasal transsphenoidal approach. The same surgical team performed all procedures.

The study protocol was approved by the Ethics Committee of Shandong Medical and Pharmaceutical University Hospital and conducted in accordance with relevant guidelines and regulations (No. LW-126 [2023]). The requirement for informed consent was waived by the Ethics Committee due to the retrospective nature of the study and the use of anonymized data.

### 2.2. Surgical procedure

**Figure 1.**
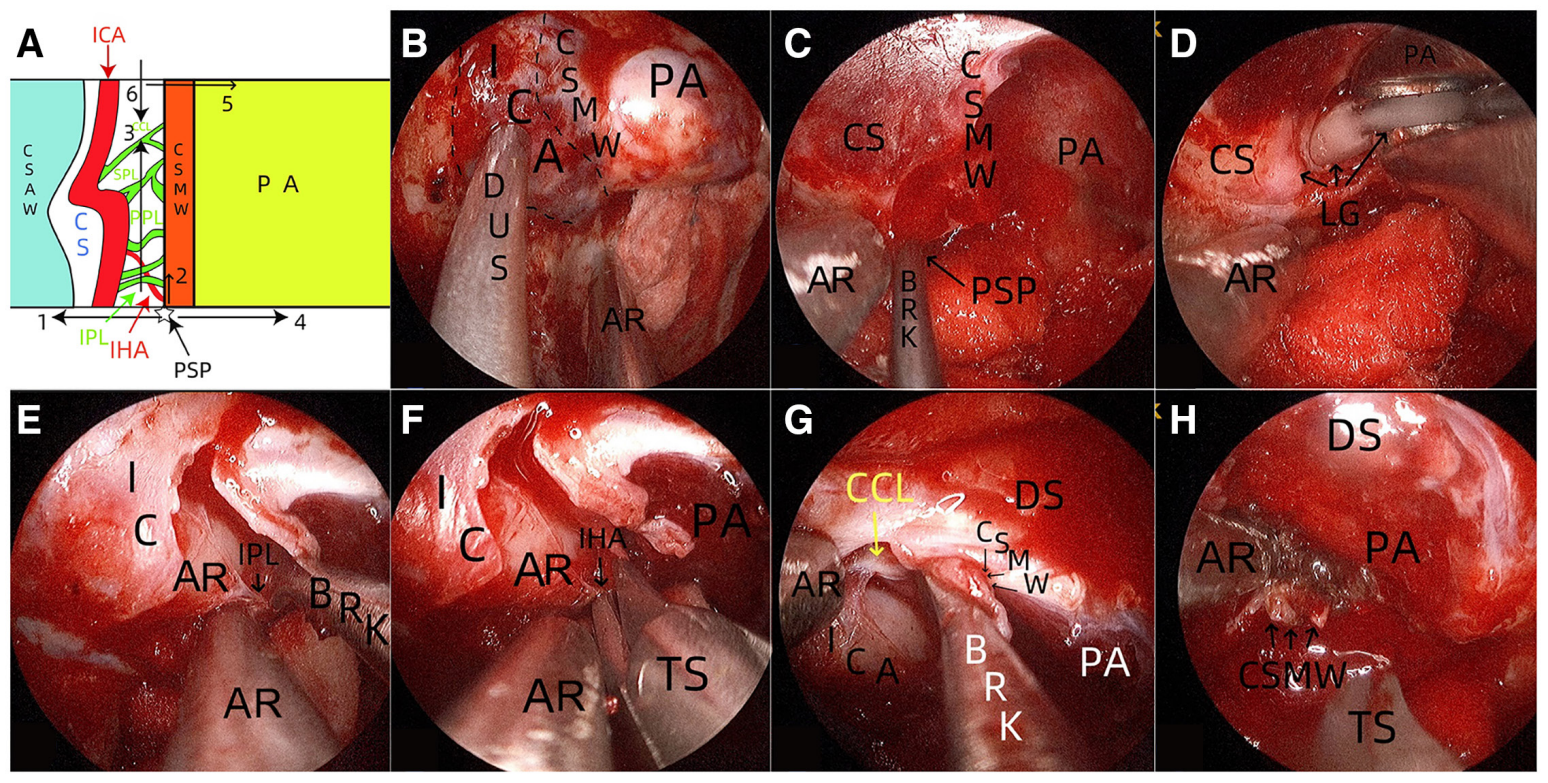
(A–H) Surgical procedure for resecting the functional pituitary adenoma and cavernous sinus medial wall. AR = aspirator, BRK = blunt right-angled knife, CCL = caroticoclinoid ligament, CS = cavernous sinus, CSAW = cavernous sinus anterior wall, CSMW = cavernous sinus medial wall, DS = diaphragma sellae, DUS = Doppler ultrasonography, ICA = internal carotid artery, IHA = inferior hypophyseal artery, IPL = inferior parasellar ligament, LG = liquid gelatin, PA = pituitary adenoma, PPL = posterior parasellar ligament, PSP = peeling starting point, SPL = superior parasellar ligament, TS = tissue scissors.

All patients underwent an endoscopic endonasal transsphenoidal approach under general anesthesia. After routine sterile preparation and nasal mucosal decongestion, a wide sphenoidotomy was performed to expose the sellar face. The bony sellar floor was thinned with a high-speed drill and removed with Kerrison rongeurs, followed by opening of the sellar dura and internal debulking of the FPA. In the CSMW resection group, a mini-Doppler probe was used to localize the internal carotid artery (ICA) and delineate its course. The cavernous sinus (CS) was entered through the inferomedial portion of the anterior wall; venous bleeding was controlled using standard hemostatic measures, and a low-pressure, limited-volume gelatin “precasting” technique was applied when necessary. Tumor extension within the CS was removed using an endoscopic 2-suction technique. The medial wall (MW) was then dissected from surrounding dural and ligamentous attachments, and the caroticoclinoid ligament (CCL) fibers were sharply divided to detach the MW from the CS; visualization of the interclinoid ligament and the dura of the oculomotor triangle served as landmarks indicating complete CCL division. Resected tumor and MW specimens were submitted for pathological examination. Multilayer skull base reconstruction was performed as indicated using dural substitute, autologous fat graft, a pedicled nasoseptal flap, and absorbable sealant materials (Fig. 1).

### 2.3. Postoperative management

Perioperative antibiotics and hemostatic agents were administered according to institutional protocols and at the surgeon’s discretion. All patients underwent brain MRI within 24 hours after surgery to evaluate early postoperative complications and the extent of resection; gross total resection was defined as the absence of residual contrast-enhancing tumor at the surgical site. Patients were assessed for cerebrospinal fluid (CSF) rhinorrhea on postoperative day (POD) 1, and nasal endoscopy was performed on POD 7 prior to removal of iodoform gauze nasal packing; additional endoscopic examinations were performed on POD 14 and POD 28 when clinically indicated. Postoperative length of stay was calculated from surgery to discharge and recorded in 0.5-day increments (durations < 12 hours counted as 0.5 day). Any postoperative epistaxis occurring between the end of surgery and hospital discharge was recorded. Biochemical cure was defined according to the Chinese expert consensus criteria: acromegaly (random growth hormone [GH], GH < 1.0 µg/L, oral glucose tolerance test if GH ≥ 1.0 µg/L, plus normalized age-/sex-adjusted insulin-like growth factor-1), prolactinoma (<15 µg/L in males and <20 µg/L in females without dopamine agonists), thyrotropinoma (normalization of free thyroid hormones with appropriately regulated thyroid-stimulating hormone and preserved/normalized other pituitary axes), and Cushing disease (early postoperative cortisol <20 µg/L within 48 hours with subsequent normalization of cortisol/urinary free cortisol/adrenocorticotropic hormone within 6 months and symptom improvement). FPAs maintaining biochemical remission for at least 6 months were considered biochemically cured. Clinical symptom relief was evaluated as improvement or resolution of disease-specific manifestations as documented in routine postoperative clinical assessments. No validated symptom-specific questionnaire or standardized scoring system was routinely administered during the study period. Intracranial infection was assessed by CSF analysis when postoperative fever or meningeal signs were present. No recurrence was defined as absence of residual or recurrent tumor on dynamic contrast-enhanced MRI at last follow-up together with endocrine findings consistent with biochemical remission and no clinical evidence of hormone hypersecretion; otherwise, recurrence was recorded. Patient-reported reduced sense of smell and nasal discomfort were collected during hospitalization and follow-up. Patients included in the final analysis had a minimum follow-up of 12 months.

### 2.4. Intraoperative data collection

Based on surgical and anesthesia records, the following intraoperative variables were extracted for both groups: operative time, intraoperative CSF leak, estimated blood loss, osseous invasion of the sellar floor, intraoperative ICA injury, intraoperative identification of an intact tumor pseudocapsule, ICA encasement, and gross total resection (confirmed on early postoperative MRI).

### 2.5. Statistical analysis

All statistical analyses were performed using SPSS software, version 29 (IBM Corp.). Propensity scores for receiving CSMW resection were estimated using a logistic regression model including age, sex, BMI, Knosp grade, hormone subtype, and tumor size/volume category. Patients in the CSMW resection group (n = 60) were matched 1:1 to patients in the non-CSMW resection pool (n = 200) using nearest-neighbor matching without replacement with a caliper of 0.2 standard deviations of the logit of the propensity score, yielding 60 matched pairs. Covariate balance before and after matching was assessed using standardized mean differences (SMDs), with SMD < 0.1 indicating adequate balance. A Love plot illustrating covariate balance before and after matching is provided in Figure S1, Supplemental Digital Content 1. Categorical variables were summarized as frequencies (percentages) and compared between matched pairs using the McNemar test (or exact McNemar test when appropriate). Continuous variables were expressed as mean ± standard deviation and compared using the paired-samples *t* test when approximately normally distributed; otherwise, the Wilcoxon signed-rank test was used. All tests were 2-sided, and a *P* value <.05 was considered statistically significant. No missing data were identified for the variables included in the analyses. Because the primary outcomes were prespecified and the secondary outcomes were considered exploratory, no formal adjustment for multiple comparisons was performed.

## 3. Results

### 3.1. Comparison of baseline characteristics between the 2 groups

After 1:1 propensity score matching, 60 patients in the CSMW resection group were matched to 60 patients in the non-CSMW resection group (matched controls). Baseline characteristics are summarized in Table 1. The 2 groups were comparable in age (56.50 ± 9.58 vs 56.89 ± 9.46, *P* = .824) and sex distribution (male: 41.7% vs 40.0%, *P* = .795). Knosp grade (*P* = .998), hormone subtype (*P* = .890), BMI (23.25 ± 2.18 vs 23.28 ± 2.76, *P* = .330), and tumor size category (GPA: 53.3% vs 53.3%, *P* = .923) did not differ significantly between the 2 groups. Comorbidities were also similar, including hypertension (55.0% vs 53.3%, *P* = .779), hyperlipidemia (51.7% vs 55.0%, *P* = .789), and diabetes (21.7% vs 28.3%, *P* = .167). Overall covariate balance after matching was acceptable, with small standardized mean differences across variables (Table 1).

**Table 1:** Baseline characteristics of patients with functioning pituitary adenomas after 1:1 propensity score matching (60 matched pairs; non-CSMW group matched from a pool of 200 patients).

Variable	Category	CSMW resection group (n = 60)	Non-CSMW resection group (n = 60, matched)	SMD	*P* value
Age (yr)		56.5 ± 9.58	56.89 ± 9.46	−0.041	.824
Sex	Male	25 (41.7%)	24 (40.0%)	0.034	.795
	Female	35 (58.3%)	36 (60.0%)	−0.034	
Knosp grade	1	8 (13.3%)	9 (15.0%)	−0.048	.998
	2	8 (13.3%)	9 (15.0%)	−0.048	
	3	38 (63.3%)	37 (61.7%)	0.034	
	4	6 (10.0%)	6 (10.0%)	0.000	
Hormone subtype	GH	23 (38.3%)	25 (41.7%)	−0.068	.890
	TSH	2 (3.3%)	2 (3.3%)	0.000	
	PRL	31 (51.7%)	28 (46.7%)	0.100	
	ACTH	4 (6.7%)	6 (10.0%)	−0.121	
BMI (kg/m^2^)		23.25 ± 2.18	23.28 ± 2.76	−0.012	.330
Hypertension	Yes	33 (55.0%)	32 (53.3%)	0.033	.779
	No	27 (45.0%)	28 (46.7%)	−0.033	
Hyperlipidemia	Yes	31 (51.7%)	33 (55.0%)	−-0.067	.789
	No	29 (48.3%)	27 (45.0%)	0.067	
Diabetes	Yes	13 (21.7%)	17 (28.3%)	−0.154	.167
	No	47 (78.3%)	43 (71.7%)	0.154	
Tumor size category	GPA	32 (53.3%)	32 (53.3%)	0.000	.923
	PMAA	28 (46.7%)	29 (48.3%)	−0.033	

Propensity scores were estimated using age, sex, BMI, Knosp grade, hormone subtype, and tumor size category; 1:1 nearest-neighbor matching without replacement was performed. Balance was assessed using SMD, with SMD < 0.1 indicating adequate balance. Categorical variables were compared using the chi-square test or Fisher exact test, as appropriate; continuous variables were compared using the independent-samples *t* test.

ACTH = adrenocorticotropic hormone, BMI = body mass index, CS = cavernous sinus, CSMW = cavernous sinus medial wall, GH = growth hormone, GPA = giant pituitary adenoma, PMAA = pituitary macroadenoma, PRL = prolactin, SMD = standardized mean difference, TSH = thyroid-stimulating hormone.

### 3.2. Comparison of intraoperative indicators between the 2 groups

Intraoperative indicators for the propensity score-matched cohort (60 matched pairs) are summarized in Table 2. Operativetime was slightly longer in the matched non-CSMW resection group than in the CSMW resection group (3.57 ± 0.57 vs 3.35 ± 0.62 hour, *P* = .008). Intraoperative CSF leak (*P* = .832), estimated blood loss (111.33 ± 118.23 vs 114.00 ± 117.92 mL, *P* = .901), osseous invasion of the sellar floor (*P* = 1.000), and intraoperative intravenous fluid volume (2439.17 ± 1116.29 vs 2284.17 ± 927.02 mL, *P* = .378) did not differ significantly between groups. Intraoperative ICA injury occurred in 1 patient (1.7%) in the CSMW resection group and 7 patients (11.7%) in the non-CSMW resection group (*P* = .070). ICA encasement was more frequent in the CSMW resection group (20.0% vs 0.0%, *P* < .001).

**Table 2:** Intraoperative information in the propensity score-matched cohort.

Variable	CSMW resection group (n = 60)	Non-CSMW resection group (matched, n = 60)	*P* value
Operative time (h)	3.35 ± 0.62	3.57 ± 0.57	.008
Intraoperative CSF leak	40 (66.7%)	42 (70.0%)	.832
Estimated blood loss (mL)	111.33 ± 118.23	114.00 ± 117.92	.901
Osseous invasion of the sellar floor	5 (8.3%)	6 (10.0%)	1.000
Intraoperative ICA injury	1 (1.7%)	7 (11.7%)	.070
Intraoperative intravenous fluid volume (mL)	2439.17 ± 1116.29	2284.17 ± 927.02	.378
Intact tumor pseudocapsule identified intraoperatively	0 (0.0%)	0 (0.0%)	1.000
ICA encasement	12 (20.0%)	0 (0.0%)	<.001
Intraoperative total tumor resection (surgeon’s assessment)	60 (100.0%)	60 (100.0%)	1.000

Data are presented as mean ± standard deviation or n (%). Continuous variables were compared using paired *t* tests; categorical variables were compared using McNemar exact tests.

CSF = cerebrospinal fluid, CSMW = cavernous sinus medial wall, ICA = internal carotid artery.

### 3.3. Postoperative complications and outcomes

**Figure 2.**
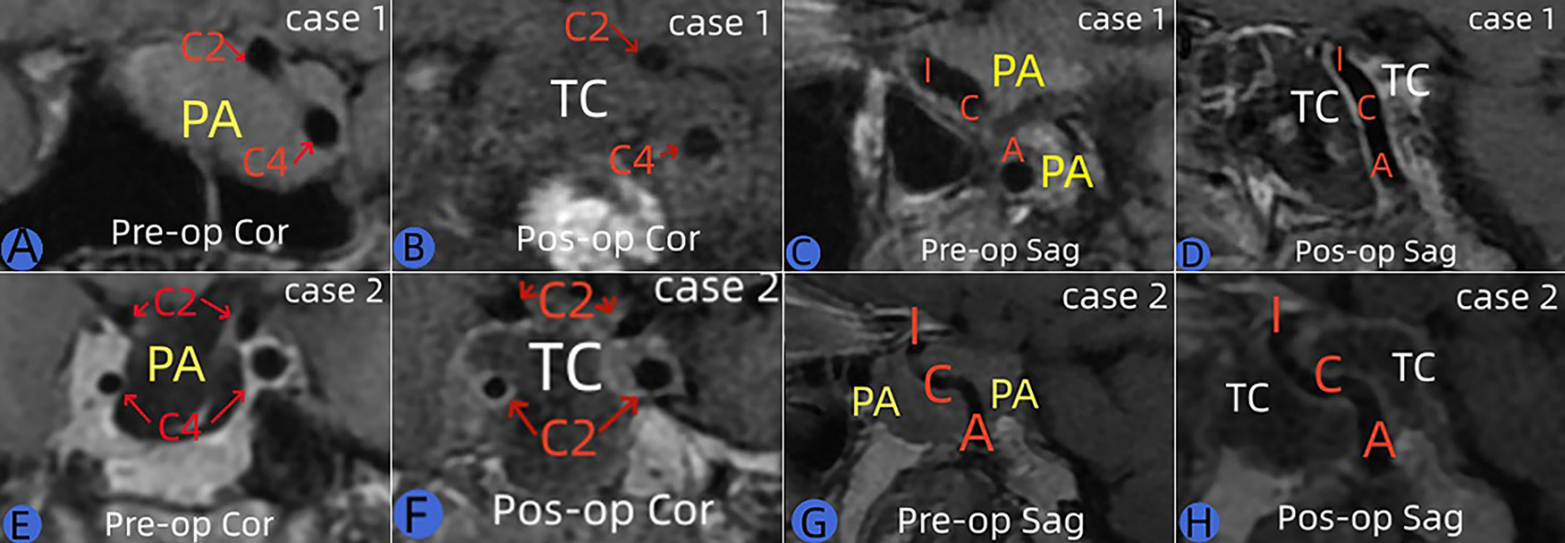
Preoperative and postoperative magnetic resonance imaging for 3 patients who underwent tumor excision combined with resection of the cavernous sinus medial wall. (A–D) Case 1. (E–H) Case 2. C2 = C2 segment of the ICA, C4 = C4 segment of the ICA, Cor = coronal view, ICA = internal carotid artery, Pos-op = postoperative, Pre-op = preoperative, PA = pituitary adenoma, Sag = sagittal view, TC = tumor cavity.

Based on the propensity score-matched cohort (60 matched pairs), the biochemical cure rate was significantly higher in the CSMW resection group than in the matched non-CSMW resection group (85.0%, 51/60 vs 63.3%, 38/60; *P* = .005). Clinical symptom relief was also higher in the CSMW resection group (83.3%, 50/60) than in the matched non-CSMW resection group (58.3%, 35/60; *P* = .002). The gross total resection rate was significantly higher in the CSMW resection group (95.0%, 57/60) compared with the matched non-CSMW resection group (83.3%, 50/60; *P* = .025). Representative preoperative and postoperative MRI images are shown in Figure 2. In contrast, tumor recurrence was significantly lower in the CSMW resection group (3.3%, 2/60) than in the matched non-CSMW resection group (15.0%, 9/60; *P* = .029). Postoperative CSF leakage (*P* = .009) and intracranial infection (*P* = .009) were also less frequent in the CSMW resection group. Length of postoperative hospital stay (*P* = .078), postoperative epistaxis (*P* = .506), reduced sense of smell (*P* = .529), and nasal discomfort (*P* = .509) did not differ significantly between the 2 groups (Table 3).

**Table 3:** Efficacy outcomes, safety outcomes, and postoperative nasal outcomes in the propensity score-matched cohort (60 matched pairs).

Outcome	CSMW resection group (n = 60)	Non-CSMW resection group (matched, n = 60)	*P* value
Efficacy outcomes			
Biochemical cure (sustained ≥6 mo)	51 (85.0%)	38 (63.3%)	.004
Clinical symptom relief	50 (83.3%)	35 (58.3%)	.002
Gross total resection (MRI-defined)	57 (95.0%)	50 (83.3%)	.045
Tumor recurrence	2 (3.3%)	9 (15.0%)	.039
Safety outcomes			
Postoperative CSF leak	2 (3.3%)	11 (18.3%)	.004
Intracranial infection	2 (3.3%)	11 (18.3%)	.004
Postoperative epistaxis	1 (1.7%)	2 (3.3%)	.506
Other postoperative outcomes			
Postoperative hospital stay (d)	6.68 ± 1.80	7.67 ± 3.57	.078
Reduced sense of smell	3 (5.0%)	4 (6.7%)	.529
Nasal discomfort	2 (3.3%)	4 (6.7%)	.509

Data are presented as n (%) unless otherwise indicated.

CSF = cerebrospinal fluid, CSMW = cavernous sinus medial wall, MRI = magnetic resonance imaging.

### 3.4. Pathological analysis

**Figure 3.**
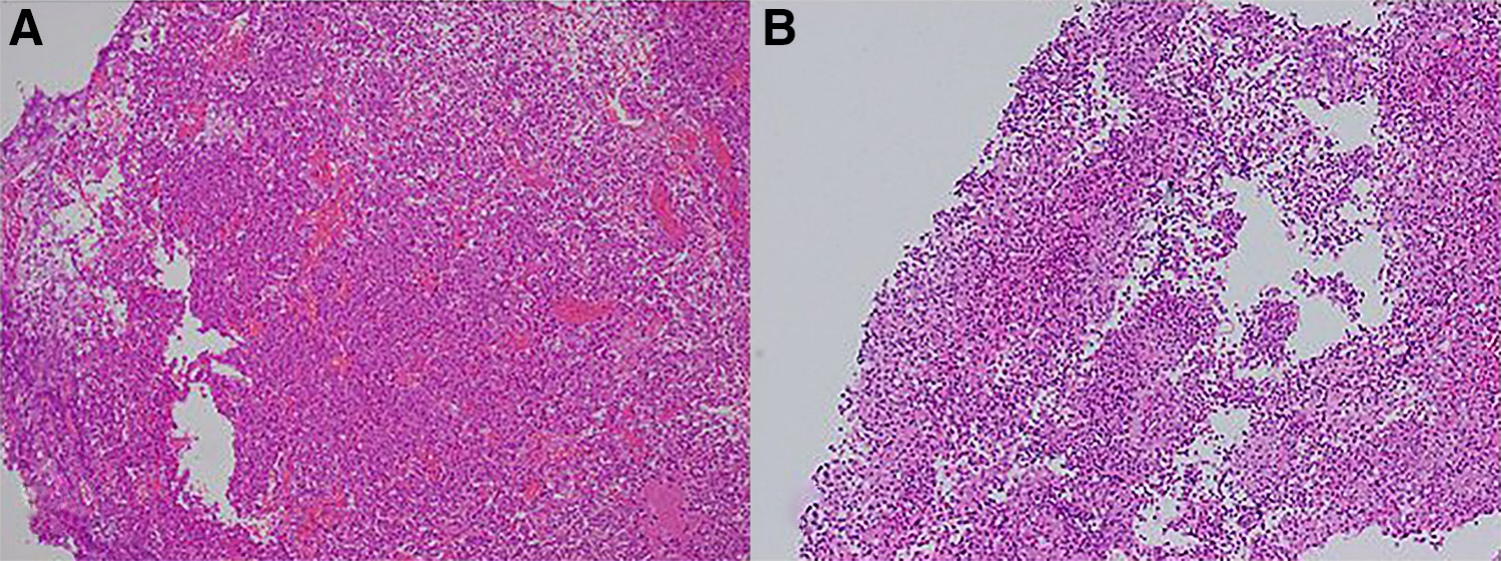
Postoperative pathological analysis. (A) (Left medial wall of the cavernous sinus) Tumor tissue is identified in the submitted specimen (100×); (B) (right medial wall of the cavernous sinus) tumor tissue is identified in the submitted specimen (100×).

Among the 60 patients in the CSMW resection group, the cavernous sinus medial wall was resected unilaterally in 58 cases and bilaterally in 2 cases, yielding a total of 62 medial wall specimens. Tumor cells were identified in the resected medial wall in 52 patients, corresponding to a patient-level detection rate of 86.7% (52/60) (Fig. 3).

### 3.5. Adjusted primary and secondary outcomes in the matched cohort

**Figure 4.**
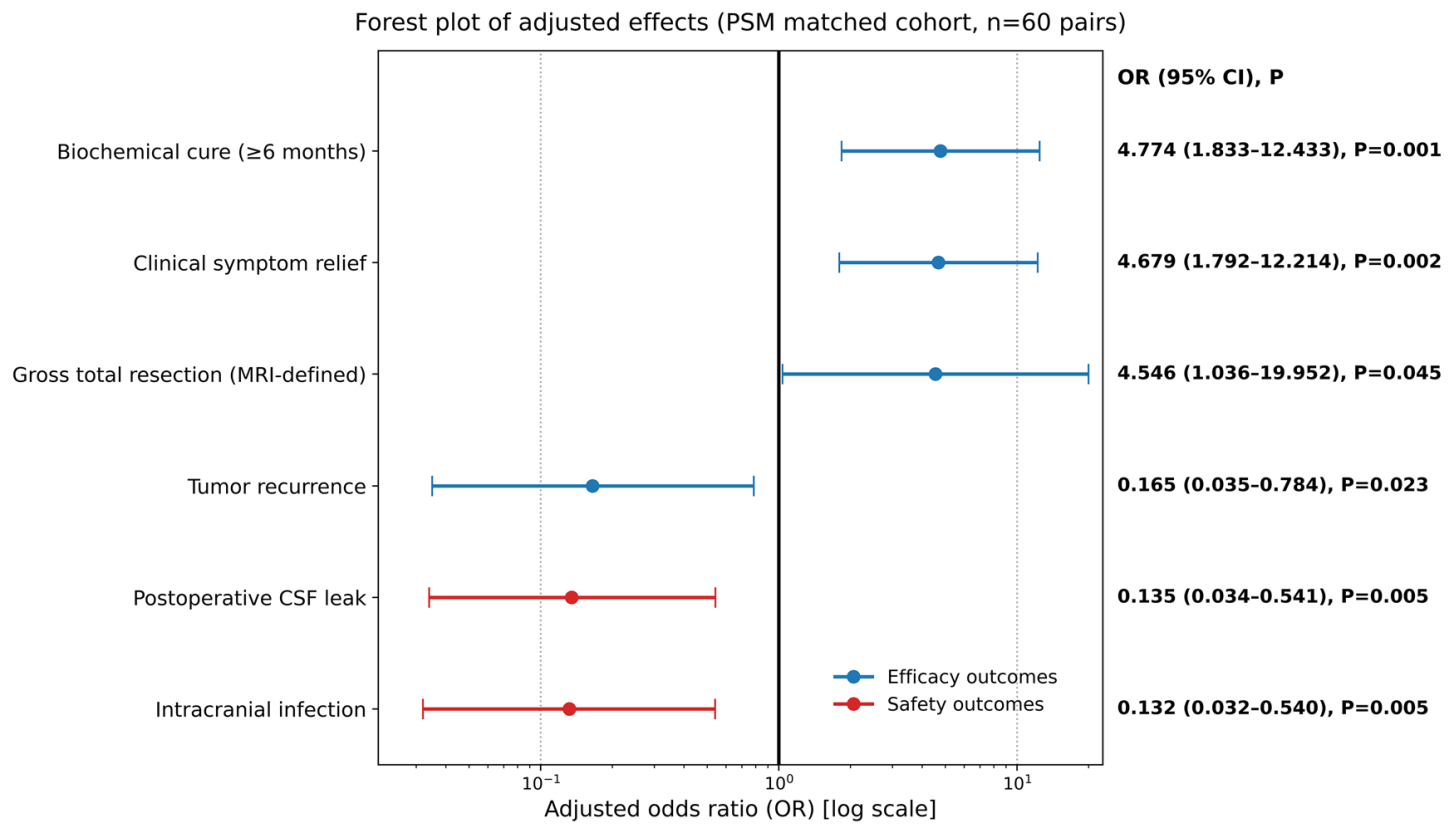
Forest plot of adjusted odds ratios for efficacy and safety outcomes in the propensity score-matched cohort. Points indicate adjusted odds ratios, and horizontal error bars represent 95% confidence intervals. CI = confidence intervals, CSF = cerebrospinal fluid, MRI = magnetic resonance imaging, OR = odds ratio, PSM = propensity score-matched.

In the propensity score-matched cohort (60 matched pairs), the CSMW resection group achieved a significantly higher rate of sustained biochemical cure (≥6 months) than the non-CSMW resection group (85.0% [51/60] vs 61.7% [37/60], unadjusted *P* = .005). After further adjustment for age, sex, BMI, and Knosp grade, CSMW resection remained independently associated with sustained biochemical cure (adjusted OR [ORa] = 4.77, 95% confidence interval [CI] 1.83–12.43; adjusted *P* = .001) (Table 4 and Fig. 4).

**Table 4:** Primary and secondary outcomes in the propensity score-matched cohort (60 matched pairs): unadjusted comparison and multivariable logistic regression.

Outcome	CSMW resection group (n = 60)	Non-CSMW resection group (matched, n = 60)	Unadjusted *P* value	ORa (95% CI)	Adjusted *P* value
Efficacy outcomes					
Biochemical cure (sustained ≥6 mo)	51/60 (85.0%)	38/60 (63.3%)	.004	4.774 (1.833–12.433)	.001
Clinical symptom relief	50/60 (83.3%)	35/60 (58.3%)	.005	4.679 (1.792–12.214)	.002
Gross total resection (MRI-defined)	57/60 (95.0%)	50/60 (83.3%)	.057	4.546 (1.036–19.952)	.045
Key secondary: tumor recurrence	2/60 (3.3%)	9/60 (15.0%)	.039	0.165 (0.035–0.784)	.023
Safety outcomes					
Postoperative CSF leak	2/60 (3.3%)	11/60 (18.3%)	.004	0.135 (0.034–0.541)	.005
Intracranial infection	2/60 (3.3%)	11/60 (18.3%)	.004	0.132 (0.032–0.540)	.005

Data are presented as n (%). Unadjusted *P* values correspond to the matched-pair comparisons reported in Table 3. ORa indicates the adjusted odds ratio for the surgical approach (CSMW vs non-CSMW) estimated from logistic regression adjusted for age, sex, body mass index (BMI), and Knosp grade, with standard errors clustered by matched pair.

BMI = body mass index, CI = confidence interval, CSF = cerebrospinal fluid, CSMW = cavernous sinus medial wall, MRI = magnetic resonance imaging, ORa = adjusted odds ratio.

For key secondary efficacy endpoints, the CSMW resection group had a higher rate of clinical symptom relief than the non-CSMW resection group (83.3% [50/60] vs 56.7% [34/60], unadjusted *P* = .002), and this association persisted after multivariable adjustment (ORa = 4.68, 95% CI 1.79–12.21; *P* = .002). MRI-defined gross total resection was also more frequent in the CSMW resection group (95.0% [57/60] vs 81.7% [49/60], unadjusted *P* = .025), with a significant adjusted association (ORa = 4.55, 95% CI 1.04–19.95; *P* = .045). Tumor recurrence occurred less often in the CSMW resection group (3.3% [2/60] vs 15.0% [9/60], unadjusted *P* = .029), and multivariable analysis confirmed a lower odds of recurrence with CSMW resection (ORa = 0.16, 95% CI 0.03–0.78; *P* = .023) (Table 4 and Fig. 4).

Regarding safety outcomes, postoperative CSF leakage was less common in the CSMW resection group than in the non-CSMW resection group (3.3% [2/60] vs 18.3% [11/60], unadjusted *P* = .009) and remained significantly lower after adjustment (ORa = 0.13, 95% CI 0.03–0.54; *P* = .005). Intracranial infection showed the same between-group pattern and remained significant in the adjusted analysis (3.3% [2/60] vs 18.3% [11/60], unadjusted *P* = .009; ORa = 0.13, 95% CI 0.03–0.54; *P* = .005). ORa were estimated using multivariable logistic regression including age, sex, BMI, and Knosp grade as covariates, with robust standard errors clustered by matched pair (Table 4 and Fig. 4).

## 4. Discussion

Overall, in this propensity score-matched analysis (60 matched pairs), baseline demographics, tumor characteristics, and comorbidity profiles were well balanced between the CSMW resection and non-CSMW resection groups, supporting the comparability of the 2 cohorts. Intraoperatively, operative time was slightly longer in the matched non-CSMW group, while ICA injury was numerically more frequent in the non-CSMW group (11.7% vs 1.7%), although this difference was not statistically significant. Other operative indicators, including intraoperative CSF leak, estimated blood loss, and intravenous fluid volume, were comparable between groups. Postoperatively, however, CSMW resection demonstrated consistently superior efficacy, with higher biochemical cure, greater clinical symptom relief, and a higher MRI-defined gross total resection rate, accompanied by a lower tumor recurrence rate. Importantly, key safety outcomes also favored CSMW resection, with significantly reduced postoperative CSF leakage and intracranial infection, whereas length of stay and other sinonasal complications did not differ materially. These findings were robust in multivariable models adjusted for age, sex, BMI, and Knosp grade, in which CSMW resection remained independently associated with sustained biochemical cure and symptom relief, improved extent of resection, reduced recurrence, and lower risks of postoperative CSF leakage and intracranial infection.

At present, Professors Miranda and Gardner at the University of Pittsburgh, along with their surgical teams, are among the leading groups worldwide in various EEA procedures. In a report published by Cohen-Cohen et al in 2018, the overall biochemical cure rate of FPA achieved by tumor resection combined with cavernous sinus medial wall resection was 97.0% (34/35).^[10]^ During the same period, Nagata et al at Nagoya University proposed the concept of “occult invasion,”^[11]^ which has been highly instructive for our work. Specifically, their data underscore that histopathological examination of the resected medial wall of the cavernous sinus represents the gold standard for determining whether a PA has invaded this structure. Nishioka et al^[12]^ reported that among 18 patients with acromegaly, tumor cells were identified in the medial wall in 16 cases, yielding a positivity rate of 88.9%. Mohyeldin et al^[13]^ found that 39 of 50 resected medial wall specimens showed histopathological evidence of tumor invasion, yielding a positive predictive value of 78% for intraoperative endoscopic assessment of medial wall invasion. Dickerman and Oldfield^[14]^ reported on 15 patients with Cushing disease, 10 of whom had “occult invasion,” meaning that no apparent invasion of the medial wall was observed under the endoscope during the initial surgery. However, these patients subsequently experienced tumor recurrence and required reoperation. The rate of tumor cell positivity in resected medial wall specimens in our cohort was comparable to that reported in prior series. In our matched cohort, we did not observe significant associations between medial wall invasion and age, sex, or admission laboratory parameters, although these analyses may be underpowered.

During follow-up, the CSMW resection group achieved higher biochemical cure and clinical symptom relief rates, with a lower recurrence rate, compared with matched controls. The MRI-defined gross total resection rate was also significantly higher in the CSMW resection group. With respect to perioperative safety, intraoperative CSF leakage, estimated blood loss, and most sinonasal complications (including postoperative epistaxis and nasal discomfort) were comparable between groups, whereas operative time was slightly longer and intraoperative ICA injury was observed more frequently in the CSMW resection group. Overall, these findings suggest that adding CSMW resection may improve disease control in FPA, while maintaining an acceptable complication profile when performed in experienced hands. Although resection of the cavernous sinus medial wall is generally considered a technically demanding procedure because of its close relationship to the ICA and cranial nerves, the incidence of ICA injury remained low in our series. Several factors may have contributed to this favorable safety profile, including routine intraoperative Doppler localization of the ICA, meticulous dissection along established anatomical planes, and the extensive experience of the surgical team in endoscopic skull-base surgery. Nevertheless, careful patient selection, thorough preoperative assessment of cavernous sinus anatomy, and strict adherence to microsurgical principles remain essential to minimize neurovascular complications during CSMW resection.

Improving postoperative biochemical cure in FPA remains a major clinical challenge. In principle, this requires maximizing the extent of resection while minimizing residual hormonally active tumor tissue. Based on the available literature, several exploratory avenues merit further investigation. First, it remains to be clarified – under strict anatomical criteria and with careful risk–benefit assessment – whether extending resection to selected adjacent membranous structures (e.g., the diaphragma sellae or other surgically accessible dural interfaces) could further reduce microscopic residual disease without increasing neurovascular morbidity. Second, intraoperative margin assessment strategies warrant development, such as rapid pathological evaluation or emerging optical imaging techniques (including hyperspectral imaging reported in other surgical fields), to better delineate tumor–membrane interfaces and guide tailored resection while avoiding unnecessary tissue injury. Third, targeted fluorescence-guided visualization – analogous to approaches used for margin delineation in glioma surgery – may be a promising direction, provided that pituitary-adenoma–specific tracers with favorable safety and specificity can be identified and validated.

In this propensity score-matched cohort, adding CSMW resection was associated with improved disease control, including higher sustained biochemical cure and symptom relief, a higher MRI-defined gross total resection rate, and a lower recurrence rate after multivariable adjustment. Although cavernous sinus medial wall resection is generally considered technically demanding because of its proximity to the ICA and cranial nerves, intraoperative ICA injury remained uncommon in both groups and was numerically less frequent in the CSMW resection group, although the difference did not reach statistical significance. Routine intraoperative Doppler localization of the ICA, meticulous anatomical dissection, and extensive surgical experience may have contributed to the favorable safety profile observed in this cohort. Furthermore, major postoperative complications were not increased; conversely, postoperative CSF leakage and intracranial infection were less common, suggesting an “overall acceptable” safety profile when the technique is performed by experienced teams.

Despite these encouraging findings, several limitations of the present study should be acknowledged. First, although propensity score matching improved baseline comparability, the final matched cohort consisted of only 60 pairs, which may have limited the statistical power to detect differences in less frequent outcomes. Second, this was a retrospective single-center study, and residual confounding and selection bias cannot be completely excluded despite propensity score matching and multivariable adjustment. Third, all patients had a minimum follow-up of 12 months, which may be insufficient to fully assess long-term biochemical remission durability and late tumor recurrence. Therefore, recurrence-related findings should be interpreted with caution. In addition, clinical symptom relief was assessed based on routine clinical evaluations rather than validated patient-reported outcome measures or standardized symptom scoring systems, which may have introduced some degree of subjective assessment bias. Finally, because all procedures were performed by an experienced endoscopic skull-base surgical team, the generalizability of these findings to other institutions requires further validation. Future multicenter prospective studies with standardized surgical indications, rigorous complication adjudication, and longer follow-up are warranted to validate these findings and refine patient selection, and to explore adjunct intraoperative technologies that may further improve margin control while preserving neurovascular safety.

## 5. Conclusion

In conclusion, in this propensity score-matched cohort, adding CSMW resection to tumor excision was associated with higher sustained biochemical cure and symptom relief rates, improved extent of resection, and a lower recurrence rate in FPAs, without an apparent increase in major perioperative complications.

## Author contributions

**Conceptualization:** Mengyang Xing, Pengfei Liu.

**Formal analysis:** Jing Wang, Yingjiang Xu.

**Funding acquisition:** Mengyang Xing, Yingjiang Xu.

**Investigation:** Jing Wang, Meng Li.

**Methodology:** Meng Li.

**Project administration:** Donghai Wang, Pengfei Liu.

**Resources:** Jinxia Liu, Yingjiang Xu.

**Software:** Jinxia Liu, Qishuai Liu.

**Supervision:** Donghai Wang.

**Validation:** Qishuai Liu, Donghai Wang.

**Writing – original draft:** Mengyang Xing.

**Writing – review & editing:** Pengfei Liu.


