# Supplementary figures and images for: Endoscopic endonasal resection of the cavernous sinus medial wall for functioning pituitary adenomas: A propensity score-matched retrospective cohort study
Source: Medicine (Baltimore). 2026 Jul 3;105(27):e49633. doi: 10.1097/MD.0000000000049633 (PMC13337065; doi:10.1097/MD.0000000000049633)

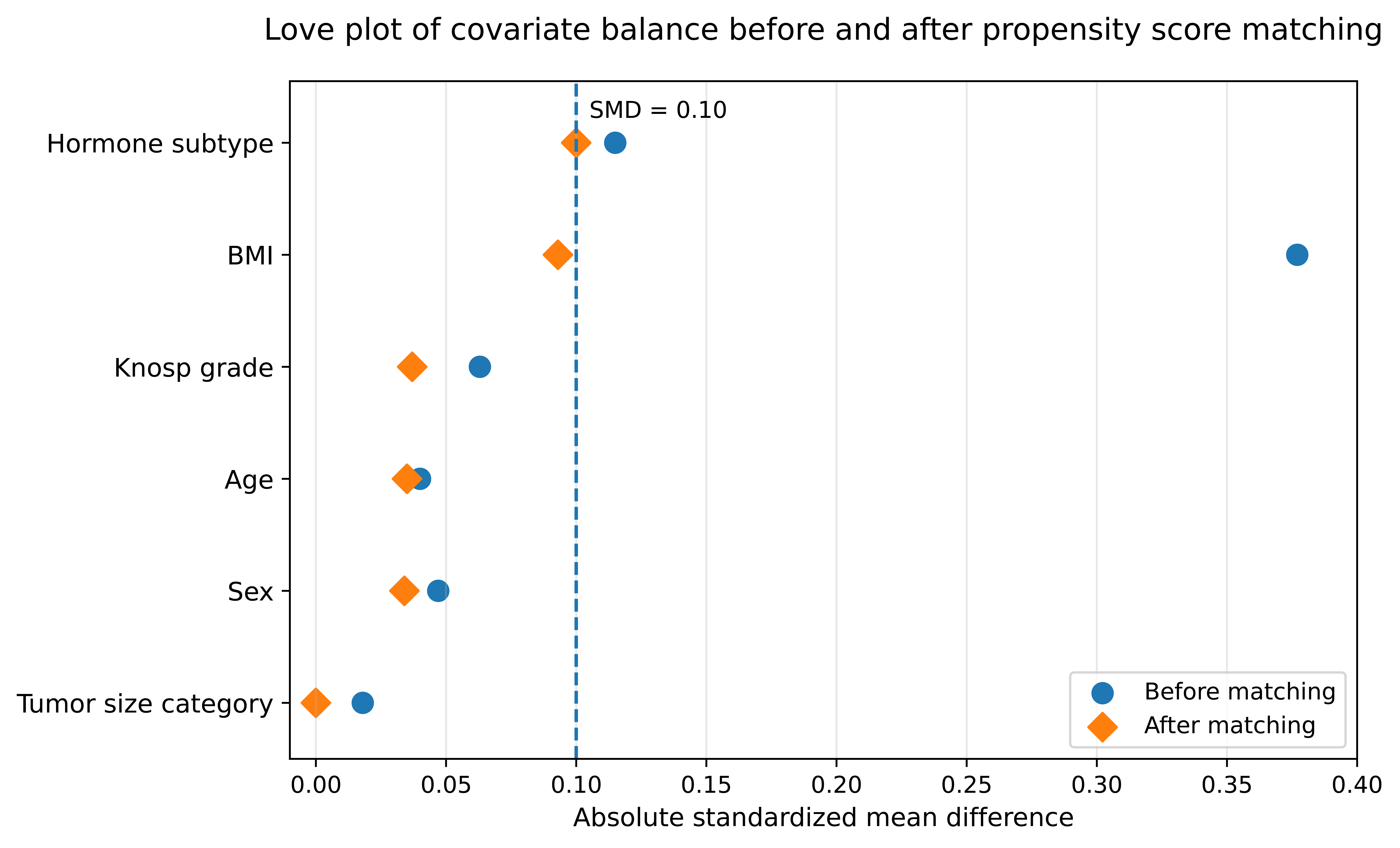

Supplement: Supplementary file 1 [file medi-105-e49633-s001.tif]
